# Strengths, weaknesses, opportunities, and threats associated with the application of artificial intelligence in connection with sport research, coaching, and optimization of athletic performance: a brief SWOT analysis

**DOI:** 10.3389/fspor.2023.1258562

**Published:** 2023-10-18

**Authors:** Billy Sperlich, Peter Düking, Robert Leppich, Hans-Christer Holmberg

**Affiliations:** ^1^ Integrative and Experimental Training Science, Institute of Sport Sciences, University of Würzburg, Würzburg, Germany; ^2^ Department of Sports Science and Movement Pedagogy, Technische Universität Braunschweig, Braunschweig, Germany; ^3^ Software Engineering Group, Department of Computer Science, University of Würzburg, Würzburg, Germany; ^4^ Department of Health Sciences, Luleå University of Technology, Luleå, Sweden; ^5^ Department of Physiology and Pharmacology, Biomedicum C5, Karolinska Institutet, Stockholm, Sweden

**Keywords:** XAI and explainable artificial intelligence, XAI, elite sport, performance, exercise science, SWOT, artifical inteligence

## Abstract

Here, we performed a non-systematic analysis of the strength, weaknesses, opportunities, and threats (SWOT) associated with the application of artificial intelligence to sports research, coaching and optimization of athletic performance. The strength of AI with regards to applied sports research, coaching and athletic performance involve the automation of time-consuming tasks, processing and analysis of large amounts of data, and recognition of complex patterns and relationships. However, it is also essential to be aware of the weaknesses associated with the integration of AI into this field. For instance, it is imperative that the data employed to train the AI system be both diverse and complete, in addition to as unbiased as possible with respect to factors such as the gender, level of performance, and experience of an athlete. Other challenges include e.g., limited adaptability to novel situations and the cost and other resources required. Opportunities include the possibility to monitor athletes both long-term and in real-time, the potential discovery of novel indicators of performance, and prediction of risk for future injury. Leveraging these opportunities can transform athletic development and the practice of sports science in general. Threats include over-dependence on technology, less involvement of human expertise, risks with respect to data privacy, breaching of the integrity and manipulation of data, and resistance to adopting such new technology. Understanding and addressing these SWOT factors is essential for maximizing the benefits of AI while mitigating its risks, thereby paving the way for its successful integration into sport science research, coaching, and optimization of athletic performance.

## Introduction

Artificial intelligence (AI) is the “theory and development of computer systems able to perform tasks that normally require human intelligence” and “makes it possible for machines to learn from experience, adjust to new inputs, and perform human-like tasks” (1). AI encompasses all forms of classical machine learning and modern artificial neural networks and through the processing of large amounts of available data (2) develop more and more human-like capabilities for decision-making and planning. The various applications of artificial intelligence (AI) are revolutionizing numerous aspects of our society (3–5), including the academic community (5) focusing on applied research relevant to sports.

This community is still in the early stages of utilizing the potential of AI (6) to maintain and improve athletic performance, prevent injuries, optimize training and assist in overall decision-making (7–9). However, as has already been carried out with other novel technologies being applied to the practice of and research on sports (10, 11), an ongoing and comprehensive understanding of the potential strengths, weaknesses, opportunities, and threats (SWOT) of AI in this context is required. To evaluate the application of artificial intelligence (AI) in sports, we executed a SWOT analysis through brief interviews with athletes, coaches, and computer science experts specialized in AI. In addition, we performed a non-systematic review of academic papers, case studies, and reports by actors in the sports industry. The broad perspective on this subject presented here is meant primarily as an aid in strategic planning, risk assessment, and resource allocation in connection with sport science research, coaching, and optimization of athletic performance.

## Strengths

### Automation of time-consuming tasks

A growing number of repetitive and time-consuming tasks, such as processing, and analysis of medical data, as well as reporting of findings can be performed by AI (12, 13). For example, language-based AI such as ChatGPT (OpenAI, CA, USA) has already demonstrated its potential to assist healthcare professionals in writing medical reports (14), although its applicability with respect to various types of sports writing remains to be established.

### Processing and analysis of large amounts of data

AI systems can process large sets of data and perform complex calculations, thereby enabling accurate and efficient analysis of (longitudinal) data on numerous athletes (9). For example, certain types of AI can already analyze the spatiotemporal behavior of soccer players in a manner that allows, among other things, automatic identification of dynamic attack formations, information which might be of value in connection with tactical training (15).

### Recognition of patterns and relationships

In connection with its rapid processing and analysis of large amounts of data, AI may reveal patterns, trends, relationships, and other insights not immediately apparent to human observers (16–20).

## Weaknesses

### Access to comprehensive, up-to-date and high-quality data at all times

When the data employed by AI fails to encompass all relevant aspects of the question being posed (6) and/or is characterized by low quality, flaws, bias, incompleteness, or unreliability, the analyses conducted and the prospective insights derived or conclusions drawn could be fallacious. Particularly worrisome in this context is the fact that the data employed to train AI models are often historical and may not always capture emerging trends, changes in techniques, strategies or rules, or individual differences that can exert a significant impact on the current situation. Similarly, changes in the situation of individual athletes, such as injuries, improvements or deterioration in form, or personal circumstances may not be adequately accounted for. To mitigate this limitation, it is essential to continuously update AI models, as well as to incorporate mechanisms that can identify emerging trends and individual variations.

### Unrecognized bias in the data on which the AI system is trained

If the training data are biased (e.g., with respect to race, sex or age), recommendations based on this data may be biased as well, as widely discussed in the medical literature (21). This situation may be exacerbated by the propagation of biases from earlier to later models of AI.

### Decision-making based on “black-box” algorithms

Some AI systems, such as artificial neural networks, can be considered “black boxes” which perform data analysis and decision-making that is un- or even counterintuitive to human brains (6). This absence of transparency in AI models can raise concerns among athletes, coaches, and other stakeholders. For instance, when an AI model is initially trained using data regarding elite athletes, but subsequently applied to non-elite or sub-elite athletes, it may introduce biases that could result in erroneous decisions and pose potential risks to athletes which would not be apparent to the humans involved, due to the opaque nature of the decision-making process.

### Over-reliance on AI technology

While AI can provide valuable insights, it should not replace human judgment entirely. Athletes and coaches should not abandon their own critical evaluations, but make the final decisions, relying on AI for support. Unfortunately, in this context recent research findings indicate the presence of a tendency towards excessive reliance on AI for decision-making, with an associated potential risk of making the wrong decision (22).

### Ethical considerations

Application of AI to sport science raises ethical concerns e.g., regarding the privacy of and consent to use the data collected, which must be addressed. In addition, over-reliance on AI may limit the personal experience and thereby hinder the personal growth of prospective trainers.

### Costs and requirements for other resources

Reliable application of AI technology to sport science may require a considerable level of expertise, significant financial investment, specialized infrastructure, and individuals who can accurately interpret the output (6). This could give teams with more resources a considerable advantage.

### Potential reduction of human interactions

As AI becomes more prevalent, humans may have to do more and more “cleaning” up and otherwise preparing data for usage by AI. This may necessitate hiring additional personnel or require regular staff members to allocate more of their time to such duties, potentially reducing the time they have available for interaction with their athletes.

## Opportunities

### Monitoring athletes both long-term and in real-time

AI allows overall individual athletic performance and relevant individual physiological variables to be analyzed both in real-time and long-term. Such feedback enables short- and long-term adjustments (23) in training load and other interventions that can help individualize and optimize both training and competitive performance. Sufficiently comprehensive data, combining video recordings with a wide variety of measurements (24), also promises to predict future performance and aid in early diagnosis of injury, followed by design, monitoring and assessment of appropriate rehabilitation.

### Prediction of risk for future injury

On the basis of their analyses of individual athletes, including their health and history of injuries, AI-based systems may aid coaches and medical staff in preventing injuries before they occur (17). In this context, artificial neural networks, decision trees and support vectors are already being used to assess risk for injury in connection with different team sports (17).

### Identification of talent

By analyzing large amounts of data concerning the determinants of performance, including physical attributes, and other potential indicators of success for individual athletes, AI can potentially help identify new talent (19, 20).

### Development of novel indicators of performance

In addition to simplifying the monitoring of indicators of performance which have been previously difficult to measure, the correct comprehensive analysis and interpretation of data on both individual and groups of athletes by AI could help reveal novel indicators of performance (25).

### More automated data- and evidence-based decision-making

The massive amounts of objective data that can be collected and analyzed by AI allow coaches and athletes to more easily make data- and, ideally, evidence-based decisions about training, competition, and coaching strategies concerning, e.g., training intensities, recovery protocols, and tactical adjustments. For instance, training plans for kickboxers generated automatically can be tailored to each individual athlete's current level of performance, period of development, and performance goals, thereby not only reducing the time required for planning, but also providing plans that were more comprehensive and personalized than those designed by an expert coach (26).

### Increased automatization of repetitive tasks, helping to improve the coach-athlete relationship

The performance of repetitive and time-consuming tasks by AI could free up more time for personal activities that improve the relationship between a coach and his athletes, which is often regarded as a key factor in the effectiveness of training (27).

### More collaboration and data sharing

AI-powered platforms and systems have the potential to facilitate collaboration and other types of interactions between sport scientists, coaches, and athletes, enabling easy sharing of data, insights, and guidelines for best practices.

## Threats

### Risks to data privacy

The collection, storage and analysis of sensitive data on individual athletes by AI can expose organizations to privacy breaches, cyber-attacks, or other forms of unauthorized access. Thus, robust data protection is required. In addition, there is the possibility that the company managing the AI system may utilize personal data for the purpose of enhancing their models, which may lead to unintentional exposure of this data to third-party users.

### Legal and regulatory challenges

The incorporation of AI into sport science raises a number of legal and regulatory concerns, such as ownership of data and copyrights, liability for deleterious consequences of AI-based decisions, and compliance with prevailing regulations concerning data protection. To address such challenges, the European Parliament recently issued a resolution aimed at legislative regulation of AI (28).

### Data integrity and manipulation

For a variety of reasons, malicious actors may attempt to manipulate the data employed by AI systems, even introducing false information, actions commonly referred to as adversarial attacks and which can alter the decisions arrived at. One such example would be manipulating the camera of a self-driving car so that it overlooks red traffic lights (29). In a similar manner, sports data could be altered by competitors to cause the opposing team to, for example, adopt suboptimal tactics.

### Deleterious social and psychological impact on athletes

Usage of AI in sport science may impair the psychological well-being of athletes, leading, for example, to performance anxiety, pressure to conform to AI-generated recommendations, and less trust in the coach. Communication and psychological aspects of the relationships between athletes, coaches and the support staff may be damaged.

### Resistance to adopting AI

As with any new technology, integration of AI into sports science may face resistance (6). There can be many reasons for such resistance and these need to be assessed employing, e.g., the technology acceptance framework (30) to survey coaches, athletes and other stakeholders.

## Limitations and future research

One limitation of the present study lies in the fact that, although our conclusions are consistent with those of both other SWOT analyses and non-systematic approaches in the realm of sports science (10, 11), we did not employ a systematic approach to the identification of strengths, weaknesses, opportunities, and threats. Consequently, we cannot guarantee the comprehensiveness of the information provided.

Furthermore, due to the continuous rapid development of artificial intelligence, new strengths, weaknesses, opportunities, and threats may emerge, while some of those discussed here may become less prominent. Future research in this area should aim to assess the application of AI to various aspects of sports science research, coaching, and optimization of athletic performance, utilizing an interdisciplinary approach involving professionals in handling and analyzing data, exercise and training of athletes, and ethical and social issues, among others.

In addition, there is a pressing need to educate sports practitioners in order to ensure that they implement AI properly and optimally, capitalizing on its strengths and opportunities, while mitigating potential threats and weaknesses. In this context, one branch of AI, i.e., Explainable Artificial Intelligence (XAI), is attempting to design machine learning architectures that are more transparent, interpretable, and accountable to human users (so-called “White” or “Glass” as opposed to “Black-Boxes”) (31). In situations where decision-making can exert immediate and long-lasting impacts, such transparency and interpretability are crucial. Understanding the rationale behind analyses performed by AI—including analyses of data on a playeŕs performance, prediction of injury, or optimization of game strategy—enables more informed and ethical usage of this technology, while at the same time promoting trust in its analyses.

## Summary

**Figure 1:**
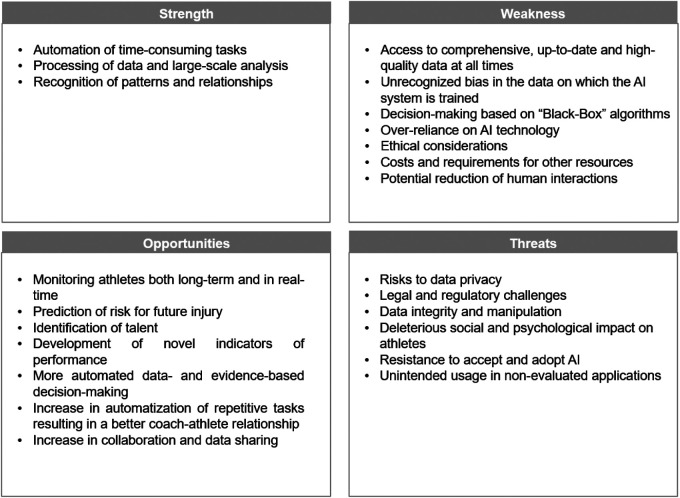
Strengths, weaknesses, opportunities, and threats associated with the use of artificial intelligence in sports science.

The use of AI in sports science research, coaching and athlete performance faces various strengths, weaknesses, opportunities, and threats, as summarized in Figure 1.

AI offers strengths such as automating repetitive and/or time-consuming tasks, performing large-scale analyses, recognizing patterns, predicting future events, and identifying talent. It can help optimize training, prevent injuries, and enhance decision-making. However, there are also weaknesses and threats to consider. The weaknesses include the need for high-quality data, a limited ability to interpret complex sport-specific situations, lack of human intuition, ethical considerations, biases, limited adaptability, costs, and lack of interpretability. Threats include risks to data privacy, legal and regulatory challenges, the integrity and manipulation of data, overreliance on AI, a negative psychological impact on athletes, technological limitations and biases, resistance to change, and overemphasis on quantitative data. It is crucial to address these weaknesses and threats for optimal usage of the strengths and opportunities offered by AI in connection with sports science research, coaching, and the optimization of athletic performance.

## Data Availability

For the original contributions presented in the article, further inquiries can be directed to the corresponding author.
